# Healthy or Unhealthy on Sale? A cross-sectional study on the proportion of healthy and unhealthy foods promoted through flyer advertising by supermarkets in the Netherlands

**DOI:** 10.1186/s12889-015-1748-8

**Published:** 2015-05-06

**Authors:** Eva AH Ravensbergen, Wilma E Waterlander, Willemieke Kroeze, Ingrid HM Steenhuis

**Affiliations:** Department of Health Sciences, Faculty of Earth and Life Sciences, VU University Amsterdam, De Boelelaan 1085, Amsterdam, 1081 HV The Netherlands; National Institute for Health Innovation, School of Population Health, University of Auckland, Private Bag 92019, Auckland Mail Centre, Auckland 1142, Auckland, New Zealand

**Keywords:** Supermarkets, Store flyers, Promotions, Food, Healthy eating

## Abstract

**Background:**

It is generally assumed that supermarkets promote unhealthy foods more heavily than healthy foods. Promotional flyers could be an effective tool for encouraging healthier food choices; however, there is a lack of good-quality evidence on this topic. Therefore, the aim of this study was to determine the proportions of healthy and unhealthy foods on promotion in Dutch supermarket flyers.

**Methods:**

Supermarket food promotions were assessed using the weekly promotional flyers of four major Dutch supermarkets over a period of eight weeks. All promotions were evaluated for healthiness,

price discount, minimum purchase amount, product category and promotion type. The level of healthiness consists of a ‘healthy’ group; products which have a positive effect on preventing chronic diseases and can be eaten every day. The ‘unhealthy’ group contain products which have adverse effects on the prevention of chronic diseases. Data were analysed using ANOVA, independent t-tests and chi-square tests.

**Results:**

A total of 1,495 promotions were included in this study. There were more promotions in the unhealthy category; 70% of promotions were categorised as unhealthy. The price discount was greater for the healthy promotions (mean 29.5%, SD 12.1) than for the two categories of unhealthy promotions (23.7%, SD 10.8; 25.4%, SD 10.5, respectively), a tendency which was mainly due to discounts in the fruit and vegetables category. To obtain the advertised discount, a significantly higher number of products had to be purchased in the unhealthy category than in the healthier categories. Promotions in the category meat, poultry and fish category occurred frequently.

Compared to traditional supermarkets, discounter supermarkets had higher percentages of unhealthy food discounts, lower discount levels and lower minimum purchase amounts.

**Conclusion:**

This research confirmed that unhealthy foods are more frequently advertised than healthier foods in Dutch supermarket flyers. Moreover, consumers had to buy more products to achieve the discount when the promotion was categorized as unhealthy, providing extra incentive for buying additional unhealthy products. Future research should explore the proportion of healthy and unhealthy food discounts in relation to supermarkets’ total product range, to determine if unhealthy products are over-represented in promotions or if there are more unhealthy products stocked in supermarkets overall. The findings of this study provide an important basis for future intervention and policy development aiming to achieve healthier supermarket environments.

## Background

Overweight and obesity continue to be growing problems, both in developed and developing countries. The percentage of obese adults is increasing, and over a billion people worldwide above the age of twenty years were overweight in 2008 [1]. In the Netherlands, the number of overweight adults has increased by more than 40% over the past 30 years. In 2010, 50% of males and 40% of females were overweight, and 10.2% of men and 12.6% of women were obese [2]. This has an adverse effect on the health of Dutch people: obese adults lose an average of 3.0 life-years and 5.1 healthy life-years (Health Adjusted Life Expectancy) [3,4]. Overweight and obesity are partly the results of a ‘westernized’ lifestyle, which includes excessive calorie intake in combination with a sedentary lifestyle [5,6]. It is increasingly recognized that an ‘obesogenic’ environment (e.g., an environment that promotes unhealthy eating habits and lifestyles), contributes to the development of overweight and obesity [7,8]. According to the Analysis Grid for Elements Linked to Obesity (ANGELO) framework developed by Swinburn [9], the environment can be divided into four categories: the ‘physical environment’, which includes the availability of products and the ease of preparation; the ‘economic environment’, which includes the costs related to food and physical activity; the ‘political environment’, which includes laws and regulations; and the ‘socio-cultural environment’, which encompasses attitudes and beliefs. Supermarkets are key players in the several environmental components of Dutch shoppers: 77% of all food purchases in the Netherlands are made in supermarkets [10]. Supermarkets use different components of the marketing mix (price, product, place and promotion) to influence what people buy [9]. Price and promotion strategies have a major effect on food purchases [11], and research has shown that price promotions significantly boost sales of certain products in supermarkets [12,13]. There is also evidence that price discounts are effective in encouraging purchases of fruit and vegetables [14]. Flyers delivered door-to-door are an important tool for communicating supermarket promotions and attracting consumers to stores [15,16]. When these flyers are combined with a 15% discount, sales increase by 173% on average [15], although this increase in sales varies by brand, product and store [17]. Although it has not been proven that price promotions are effective in boosting long-term supermarket sales [13] or increasing profits [12], the distribution of flyers has a distinct purpose. On average, 89% of Dutch shoppers receive three flyers per week from supermarkets, and 83% of all these flyers are read by these consumers [15]. Through these promotions, supermarkets attempt to attract price-sensitive shoppers into their stores and boost spending by regular customers [18,19].

In-store supermarket promotions can be seen as temporary improvements in the price-value ratio of products. This improved price-value ratio can be achieved either by a temporary price reduction or by an increased volume of the product for the same price. Many products are promoted in supermarkets every week. Promotions aside, the healthy choice is often perceived by consumers as being the more expensive choice, and price has been found to be a barrier to healthier purchases [11,20,21]. Research has revealed that price discounts on easy-to-store products and products with a long shelf life are more likely to boost sales than discounts on products that have a shorter shelf life or are difficult to store [15]. However, a 25% discount on fruits and vegetables was effective in stimulating purchases in this product category [22]. Supermarkets seek to embed promotions efficiently through the use of category management, a process in which the total product range of a supermarket is broken down into discrete groups of similar or related products; these groups can be seen as small strategic business units. This approach is used to provide a framework for the evaluation of promotions and pricing in order to achieve the optimum product mix within the different product categories. Differences in pricing strategies within different product categories are not uncommon in supermarkets [23].

It is often suggested that unhealthier products are promoted more frequently than healthier products, and there is some evidence to support this. Although little research has been performed on the ratio of healthy to unhealthy promotions advertised in supermarket flyers in the Netherlands, it is clear that the food industry invests a great deal in marketing unhealthy products [24-26]. Research has shown that 80% of the food products promoted through television advertising are high-fat and high-sugar foods [27]. US research that assessed the types of foods advertised in supermarket newspapers circulars showed that front pages devote most advertising space to protein-rich foods; furthermore, advertisements do not consistently emphasize foods that support healthy weight [28]. Also, a study of all price promotions run by British supermarkets showed that promotions of fatty and sugary foods outnumbered those of fruit and vegetables by more than two to one (no distinction was made between promotions advertised in flyers and those that were offered only in stores) [29]. However, international research has shown that in Dutch supermarkets only half of all checkout displays featured snack foods or soft drinks, and that there are relatively few unhealthy products promoted in end-of-aisle displays—positive attributes compared to supermarkets worldwide [30].

Furthermore, in addition to influencing purchasing behaviour, promotions can influence consumption rates. Although this effect is highly complex and differs by type of promotion, product category, and the characteristics of the food product and the consumer, there is evidence that people consume more of the products they purchase on promotion [23,31]. Given this information, increasing the number of healthy products advertised in store flyers could be a strategy for promoting healthier eating. In general, however, there is a lack of good quality evidence on the ratio between healthy and unhealthy food promotions. First and foremost, it would be useful to know whether there is a genuine difference between the characteristics of promotions of healthy and unhealthy food. The main aim of this study, therefore, was to determine the proportion of healthy and unhealthy promotions advertised in store flyers from supermarkets in the Netherlands. We assessed the following factors: (1) differences in price discounts between healthy and unhealthy promotions; (2) differences in minimum purchase amounts between healthy and unhealthy promotions; and (3) the frequency of promotions in the various product categories. The hypothesis was that unhealthy foods are more frequently promoted in store flyers than healthy foods. Secondly, it was hypothesized that a higher discount is available on unhealthy foods than healthy foods.

## Methods

This cross-sectional study was conducted in four supermarket chains in the Netherlands. Data were collected over an eight-week period, and a total of 32 printed supermarket flyers were used for this study. (We also examined online promotions, but these were the same as those listed in the printed flyers and were therefore not included in this study.). These flyers contained 1,818 in-store promotions, of which 1,515 (83.3%) were promotions for food products.

### Selection

We started by selecting the supermarkets to be analysed in this study according to their market shares (in the year 2010), with the aim of choosing the top four supermarkets [10]. We initially selected four supermarkets, which together accounted for 58.5% of the market share. An additional requirement was that the supermarkets should distribute a printed weekly flyer, a paper copy of which was available in-store and/or was home delivered. One supermarket was excluded as a result of this criterion. Another requirement was that the flyer had to be identical in every region of the Netherlands. Finally, the flyers had to include sales promotions. One supermarket was excluded as a result of this criterion. After these exclusions, we selected the market leader and the supermarkets in the fourth, fifth and sixth positions in terms of market share. These supermarkets had, respectively, market shares of 34%, 12.5%, 6.0% and 5.6% in 2010, making a total of 56.5% [10]. One of the included supermarkets was a discounter. A discounter was defined as a supermarket with prices lower than the typical market value; these supermarkets focus on price rather than service, display, or choice [32].

### Measures

We started by recording all promotions advertised in the supermarket flyers. If a promotion consisted of multiple products, this was counted as one promotion. For example, one promotion for sliced Dutch cheese consisted of different types of cheese, such as reduced fat, full fat, mature or with chives, and despite the different types involved, this was categorised as a single promotion: sliced Dutch cheese. We followed the rule that the depiction and definition of the promotion advertised in the supermarket flyer determined the number of products included in one promotion.

### Healthiness of the promotions

The promotions advertised in the supermarket flyers were scored for healthiness. Healthiness was assessed according to the Dutch ‘Guidelines for Food Choice 2011’, which were published by the Health Council of the Netherlands and were partly based on the nutritional guidelines of the World Health Organization [33]. These guidelines apply a three-way system for assessing the healthiness of products. The three categories include ‘preference products’, ‘occasional products’ and ‘products for exceptional cases’ (also referred to as ‘rare products’). Preference products have a positive effect on preventing chronic diseases and can be eaten every day (e.g., apples, beef tartar). Products in the occasional group can still make up part of a healthy diet but should be eaten less frequently and in smaller amounts compared to the preference group (e.g., wheat bread, gingerbread, high-fibre cornflakes). Products in the rare group contain nutrients which have adverse effects on the prevention of chronic diseases. Products in this category contain higher levels of saturated fat, energy and/or salt compared to the other categories, and should be eaten only in rare circumstances (e.g., white bread, crisps, chocolate cookies) [34]. Table 1 shows an excerpt of the three levels of healthiness and associated products. The promotions we studied were classified into these three groups according to their levels of saturated fat, trans fat, fibre, sodium and energy, which varied between product categories. Here, healthiness was based on the ‘promotion level’ rather than on product level. In some cases, promotions contained products in more than one health category; such promotions were classified into the least healthy of these categories. For example, one cheese promotion included both reduced fat and full fat Dutch cheese (respectively classified as preference and rare foods); in this case, the cheese promotion was allocated to the rare group. The nutritional values of the products studied were derived from the Dutch Food Composition Table [35], which contains data on energy and 47 nutrients in 2,080 foods. Products not included in the database were assigned nutritional values according to those listed on product packaging [35]. The Guidelines for Food Choice list products which are often consumed by the Dutch population. For products on sale which were not listed in this table, additions to the Guidelines were used. These additions included a table with nutritional values, which can be used to allocate products to levels of healthiness in the same way the commonly used products are classified (the amount of saturated fat, sodium, fiber, and energy were taken into account).

**Table 1:** **Excerpt from the ‘Guidelines for Food Choice 201**1’ [34]

**Product category**	**Preference**	**Occasional**	**Rare**
Bread (substitutes), cereals	Rye bread, whole grain crisp bread, bread, whole wheat bread	Brown bread, bun, multigrain bread, oatmeal, muesli with fruit	White bread, croissant, rusk, chocopops, frosties, cornflakes
Cheese	Low fat cheese, mozzarella, cottage cheese, fresh goat cheese, diary spread light	Camembert, cream cheese, diary spread	Full fat cheese, cheddar, cream cheese, gorgonzola, blue cheese
Diary	Skimmed milk, low fat yoghurt, buttermilk	Semi-skimmed milk, low fat custard	Full fat milk, pudding, yoghurt drink, full fat yoghurt, custard
Starch products	Boiled potatoes, baked potatoes, whole wheat pasta, brown rice, couscous	Mashed potatoes, multigrain rice	French fries, fried potatoes, boiled cassava, regular pasta, white rice
Spreads	Lean frankfurter sausage, chicken, vegetarian pate, roast beef		Ham, bacon, loin roast, sausage, smoked meat, liver pate
Vegetables	All vegetables boiled or raw, frozen or canned vegetables (without additives)	Vegetable puree, pickled peppers, tomato juice without salt	Vegetables with cream, olives, pickles, onions, tomato juice with salt

Healthiness was determined for all products with the exception of food specially designed for babies and toddlers up to 36 months of age, since they have different food guidelines which were not included in the Guidelines for Food Choice (N = 9 promotions) [33]. Eggs and spices (n = 11 promotions) were also excluded from the health status, since the Guidelines for Food Choice do not include these products.

### Price discount and minimum purchase amount

The price discount was measured as a percentage, per promotion. Within a multi-item promotion, it was possible that different products would qualify for a different relative price discount (e.g., all brands of custard are on sale for the special price of one euro, but the original prices of single products differed); in such cases, the percentage of discount differed as well. For these promotions, we included the average discount across the range of products. Furthermore, we recorded the minimum purchase amount for all promotions. This minimum purchase corresponded to the minimum purchase amount needed to receive the advertised price discount (e.g. 3 items for €5).

### Product categories

Promotions were classified into product categories, as shown in Table 2. These categories were based on the Dutch Guidelines for Food Choice and the categorizations used by the Dutch market leader. Promotions were placed into a product category based primarily on intended use, the origin of the product, and/or the positioning selected by the manufacturer. Promotions consisting of multiple products could involve a combination of product categories. Accordingly, these promotions were automatically placed in a separate category. For example, one promotion was for fresh Asian vegetables and seasoning (one of both products had to be purchased to receive a discount of 33%), and this promotion was placed in a special category (‘combination of categories’). Vegetables belong in the second category, while seasoning belongs in the sixth category; because of this combination, this promotion was placed in the combination of categories, the eighteenth category.

**Table 2:** **Categorization of promotions**

**Product category**	**Explanation**
1. Fruits	All fruits including processed fruits in which the total edible portion of the original product is still present in the final product, with the exception of fruit juices
2. Vegetables	All vegetables including processed vegetables in which the total edible portion of the original product is still present in the final product
3. Starch products	Potatoes, pasta, rice, legumes, potato products and other starches used for main meals
4. Meat, poultry, fish	All meats, including composite meat products, poultry, meat substitutes, meat preserves, fish and eggs; both processed and unprocessed
5. Ready to eat meals, soups, pizzas	All meals that consist of a plurality of components, which are ready to eat, including salads and pizzas
6. International, seasonings	All (meal) sauces; including meal mixes needing an addition, according to the label, of starch and/or protein source and other international products and seasonings
7. Cheese	All sorts of cheese including cheese spreads
8. Diary	All kinds of milk (substitutes), including milk with additives and processed milk
9. Bread (substitutes), cereals	All sorts of breads and baked cereals which are normally eaten with spreads, and cereals that are normally eaten with milk (products)
10. Pastry, cakes, candy, ice cream, chocolate	All sorts of sweet pastry, cakes, candy, ice cream and chocolate which are intended to be eaten as a snack between meals
11. Pretzels, crisps, snacks, nuts	All sorts of pretzels, crisps, snacks and nuts which are intended to be eaten as a snack between meals
12. Beverages, fruit juices	All beverages and juices except coffee, tea, alcoholic beverages and dairy drinks
13. Prepared meat products	All sorts of meat products which are primarily eaten as a spread
14. Spreads	All (sweet) spreads including ‘salads’ (e.g. egg salad, tuna salad) with mayonnaise designed to be spread on bread/toast; excludes cheese and prepared meat products
15. Coffee and tea	All coffee, tea and related products
16. Alcoholic beverages	All beverages with an alcohol percentage of 0.5% and higher
17. Butter, fats, oils	All fats intended for spreading on bread or for use in the preparation of food
18. Non-Food	All inedible products
19. Combination of product categories	All promotions that contain a combination of multiple product categories

### Promotion types

For this study, we differentiated between different types of promotion. The first distinction was between single-item promotions (only one product had to be bought to receive a price discount) and multi-item promotions (two or more products had to be purchased to receive a price discount). Then, single-item promotions could be subdivided into fixed promotions (no choice between products for the consumer) and self-bundling (the consumer could choose between two or more products within a certain product category or range of products). Similarly, multiple-item promotions could be subdivided into fixed promotions and self-bundling; the fixed multiple-item promotion category was applicable when two specific products had to be bought to gain a price discount, or when products were identical [36,37].

Furthermore, we recorded the use of permanent price reductions in supermarket advertising flyers. In contrast to the other promotions types, this type of promotion is not temporary, and may be the result of an ongoing price war among supermarkets in the Netherlands [38,39]. This price war, initiated by the market leader in October 2003, is in keeping with international trends in supermarket pricing strategies, and has led to strong competition between supermarkets focusing on price-based promotions [40]. In this situation, the importance of store loyalty on the part of consumers is less important. Another strategy supermarkets used in the flyers, intended to attract time-constrained shoppers, was fixed (low) pricing as part of an ‘Every Day Low Pricing’ (EDLP) strategy [19]. This strategy promises consumers low prices at all times. Since the start of the price war among supermarkets in the Netherlands, supermarkets use these ‘offers’ in combination with price discounts in their flyers [15]. Other types of offers or promotions for which it was unclear which type of promotion was involved were combined into the category ‘remaining promotions’.

### Statistical analysis

A total of 1,818 promotions were advertised in supermarket flyers during the research period, of which 1,515 promotions involved food products. 1,495 of these advertised promotions were evaluated for healthiness and included in our analysis. Measures for product categories and promotion types were used for the descriptive analysis to gain insight into the distribution of promotion types and product categories used for advertisements in the flyers. Differences in the degree of price discounts between the three healthiness categories were evaluated by one-way ANOVA. The Tukey analysis was used as a post-hoc method to determine significant differences between groups. The same statistical technique was used to measure differences in the minimum purchase amounts between the healthiness categories. We conducted sensitivity analysis to examine whether classifying bundled promotions into the unhealthier category influenced our results. Since one discount supermarket was included in this study, we tested for differences in the discount percentage, minimum purchase amount and promotions in the different healthiness categories between discount and traditional supermarkets using independent T-tests and chi-square tests. Here, the data from the promotions from the three traditional supermarkets were averaged. This average was used to measure the differences between the traditional supermarkets and the discounter. An independent T-test was performed to determine differences in the percentage discount and minimum purchase amount between the two types of supermarkets. A Chi-square analysis was then performed to determine whether there was a difference between supermarket types in the number of promotions in each health category. Analyses were conducted using the SPSS statistical software package, version 17.0. (SPSS Inc., Chicago, IL, USA).

## Results

Of all promotions, 66.7% were for products in the rare category, 29.7% were for preference products, and 3.7% were for occasional products. Table 3 shows differences in the mean discount between these healthiness categories. The highest discounts occurred in the preference group (29.5%). The lowest mean discount was observed in the occasional group (23.7%); the differences between the groups were statistically significant (p < .01). Post-hoc tests further revealed that products in the preference group had significantly higher mean discount rates than both the occasional and rare groups (p < .01). Furthermore, Table 3 shows the differences between the three healthiness categories with regard to the minimum number of products needed to purchase to obtain a price discount. The rare category had a higher minimum purchase number (mean = 1.5, SD = 0.76) than the preference group (mean = 1.3, SD = 1.26) and the occasional group (mean = 1.4, SD = 1.38). However, only the difference in the minimum purchase amount between the preference and rare groups was statistically significant (p < .001). Sensitivity analysis revealed that re-classifying bundled promotions from the unhealthier to the healthier category raised the number of promotions to 1795; 39.4% of these promotions were classified to the preference group and 55.8% to the rare group. The reclassifying of the promotions reduced the differences between the percentage discounts (28.4% to 25.9%), although the differences remained significant (p < .001).

**Table 3:** **Differences in mean discount level and minimum purchase amount between the healthiness categories**

	**% Discount**		**Minimum purchase**	
	**Mean**	**SD**	**Mean**	**SD**
**Preference**	29.5	12.1	1.26	0.51
**Occasional**	23.7*	10.8	1.38	0.55
**Rare**	25.4**	10.5	1.50**	0.76

Significant difference vs the preference group *P <0.01, **P = <0.001.

Table 4 shows that the promotions were most frequently observed in the category of meat, poultry and fish (19%), followed by the category of sweet snacks (14.3%) and then of vegetables (7.3%). Promotions were least frequent in the category of butter, fats and oils (1.2%) followed by the combination of product categories (1.5%). The highest discount rates were found in the category of fruit (38.2%), followed by starches (32.4%) and vegetables (31.7%). Looking at the minimum purchase amount required, the highest minimum purchase was in the category of beverages and fruit juices (minimum 2.1 units per promotion) (Table 4). The most commonly used promotion type was the fixed single item promotion (31.1%), closely followed by the self-bundling multi-item promotion (26.6%). Fixed (low) price (2.1%) and fixed multi-item promotion (6.1%) were used to a lesser extent.

**Table 4:** **Differences in discount frequency, average discount and minimum purchase amount per product category (measured in 2012)**

**Product categories**	**Freq**	**% of total offers**	**Average (%) discount**		**Average minimum purchase amount**	
			**Mean**	**SD**	**Mean**	**SD**
1. Fruit	68	4.5	38.1	10.8	1.15	0.35
2. Vegetables	110	7.3	31.7	10.8	1.20	0.44
3. Starches	39	2.6	32.4	14.7	1.56	0.64
4. Meat, poultry, fish	288	19.0	26.5	8.93	1.15	0.38
5. Ready-to-eat meals, soups, pizzas	82	5.4	27.1	10.0	1.70	0.84
6. International, seasoning	57	3.8	22.4	13.1	1.56	0.86
7. Cheese	68	4.5	27.6	9.8	1.31	0.62
8. Diary	78	5.1	25.2	11.8	1.51	0.65
9. Bread (substitutes), cereals	83	5.5	26.1	10.1	1.43	0.87
10. Pastry, cakes, candy, ice cream, chocolate	217	14.3	23.1	9.41	1.55	0.82
11. Snacks, crisps, pretzels, nuts	72	4.8	24.0	13.9	1.57	0.70
12. Beverages, fruit juices	70*	4.6	26.7	10.6	2.14	0.95
13. Prepared meat products	83	5.5	26.8	9.81	1.34	0.61
14. Spreads	28	1.9	20.5	10.6	1.39	0.56
15. Coffee, tea and related products	36	2.4	19.3	12.4	1.61	0.68
16. Alcoholic beverages	95	6.3	25.2	9.59	1.34	0.75
17. Butter, fats, oils	18	1.2	21.2	12.9	1.14	0.51
18. Combination of multiple product categories	21	1.5	30.8	9.30	2.05	0.48

*34% soft drinks.

### Differences between types of supermarkets

Table 5 shows the differences in mean promotion levels within the three healthiness categories between discount supermarkets and regular supermarkets. The discounters had a significantly higher number of promotions on rare products (284; equivalent to 75.9% of total promotions) than traditional supermarkets (245; equivalent to 63.3% of total promotions) (p < .001). Within the preference group, traditional supermarkets had the highest level of promotions (129; equivalent to 33.3% of total promotions); this was significantly higher than the discount supermarkets (71; equivalent to 19% of total promotions) (x^2^ = 25.82; p <0.001). Furthermore, a significantly higher rate of discounts was observed for the traditional supermarkets (28% compared to 21%, p < .001). Overall, the minimum purchase amount required in the discount supermarkets was significantly lower (1.2) than that required in traditional supermarkets (1.5) (T = 11.14; p < .001). Finally, as shown in Table 6, the discounters had a total of 85 promotions on sweet snacks, representing 22.7% of total promotions. This was relatively high when compared to those for the traditional supermarkets, which had an average of 45 promotions for sweet snacks, representing 11% of their total promotions. Notable in both supermarket types was the higher frequency of promotions for sweet snacks compared to salty snacks. Traditional supermarkets had a higher rate of promotions for the category of vegetables (41 promotions compared to 8 in discount supermarkets).

**Table 5:** **Differences in healthiness, percentage discount and minimum purchase amount between traditional and discount supermarkets**

	Healthiness***						**% Discount**		**Minimum purchase**	
	**Preference**			**Rare**						
	**Mean**	**SD**	**% of total within supermarket**	**Mean**	**SD**	**% of total within supermarket**	**Mean**	**SD**	**Mean**	**SD**
Traditional	129	26.04	33.3%	245	44.00	63.3%	27.9	12.1	1.54	0.76
Discounter	71**	-	19.0%	284**	-	75.9%	21.4	7.38	1.19**	0.46

Significant difference vs traditional supermarkets *P < .01; **< .001.

***Occasional group is disregarded because it is redundant.

**Table 6:** **Differences in product categories of promotions between traditional and discount supermarkets**

	**Traditional supermarket**		**% of total within supermarket**	**Discount supermarket**	**% of total within supermarket**
	**Mean**	**SD**			
1. Fruit	20.3	4.775	4.9	11	2.9
2. Vegetables	41.3	16.429	10.0	8	2.1
3. Potatoes, pasta, rice, legumes	10.7	0.47	2.7	7	1.9
4. Meat, fish, poultry	72.4	15.40	17.6	80	21.3
5. Meals, soups, pizza	21.4	4.026	5.1	20	5.3
6. International, seasoning	16.2	4.028	3.9	13	3.5
7. Cheese	17.3	4.181	4.2	20	5.3
8. Diary	20.3	2.451	4.9	18	4.8
9. Bread, bread substitutes, cereals	24.2	7.751	5.9	21	5.6
10. Pastry, cake, candy, ice cream, chocolate	45.5	8.308	11.0	85	22.7
11. Snacks, chips, pretzels	21.48	9.067	5.1	22	5.9
12. Drinks, juices	20.64	2.436	5.1	9	2.4
13. Meats	23.73	6.176	5.9	17	4.5
14. Spreads	8.24	3.048	2.0	7	2.1
15. Coffee, tea and belongings	12.20	2.576	2.9	1	0.3
16. Alcohol	21.80	1.743	5.4	30	8.0
17. Butter, fats, oils	5.31	2.016	1.2	5	1.3
18. Combination of product categories	9.18	3.404	2.2	0	0

## Discussion

This research confirmed the hypothesis that unhealthy foods are more frequently advertised in Dutch supermarket flyers than healthier foods. Promotions were categorised as healthy for only 29.8% of all promotions advertised. However, the price discounts were much higher for healthy promotions than for unhealthy promotions, a tendency which is mainly reflected in the categories of fruit and vegetables. Furthermore, the results showed that a significantly higher number of products categorized as rare (i.e., containing nutrients which have an adverse effect on the prevention of chronic diseases) had to be bought to obtain the advertised price discount compared to promotions on products in the preference group. Promotions were most frequently found for the category of meat, poultry and fish, followed by the category of sweet snacks. Finally, significant differences were found between traditional and discounter supermarkets. Compared to traditional supermarkets, discounter supermarkets had more promotions for unhealthy food, while offering a lower percentage discount and a lower minimum purchase amount (all were significant).

The main aim of this study was to determine the proportion of healthy and unhealthy promotions advertised through Dutch supermarket flyers. A previous study performed in the UK found twice as many price promotions in British supermarkets for fatty and sugary foods than for fruit and vegetables [29]. Our results are broadly in line with this study. However, the promotions categorized as healthy had a significantly greater price discount than unhealthier products. A greater price discount could lead to increased sales of healthy products, and could therefore be beneficial for public health [15]. However, it should be remembered that supermarkets generally have higher margins on fruit and vegetables; the gross margin, which can be as high as 63% in supermarkets, allows supermarkets to promote these products while still making a profit [41].

The higher minimum purchase amount required for promotions in the rare group indicates that rare promotions are more often advertised in multi-item promotions than single-item promotions. This means that a consumer has to buy more products to make a saving. This is unfavourable, since additional purchases are linked to higher caloric intake and therefore contribute to the problem of overweight [15,42].

Our study revealed a high frequency of promotions for sweet snacks, which is in line with previous research indicating frequent promotions for sugary or fatty foods [27]. Moreover, our results revealed more promotions in the category of meat, poultry and fish. This appears to contradict previous findings which showed that price discounts on easy-to-store products and products with a long shelf life can increase sales more effectively than price discounts on products with a shorter shelf life that are more difficult to store [15]. Nevertheless, meat, poultry and fish are generally expensive, but also much in demand, so discounts in this category could attract consumers to a supermarket.

The comparison between traditional supermarkets and discounters showed that traditional supermarkets had higher average discount levels. This can be explained by the fact that prices in discount supermarkets are kept consistently low; such supermarkets do not focus on special offers. Discounters offer a relatively large number of house brands (products particular to that supermarket chain) in their range. This results in such supermarkets stocking a smaller range of products than traditional stores, which sell other brands alongside their own house brand. All of these strategies among discounters mean that these stores have less scope for offering price discounts. Additionally, price discounting is not an important marketing technique for the discounter supermarkets [19,43]. We also found that discounters advertised fewer promotions on vegetables than regular supermarkets. This could also be the result of their smaller range of products [44]. Increasing the frequency of promotions on healthier products, including vegetables, could be an important intervention for increasing sales of healthier foods. Discount stores generally attract consumers of low socio-economic status (SES), among whom the prevalence of overweight and obesity are higher than in those of higher SES [45]. Research has shown that lower SES groups perceive financial barriers to buying healthier foods [46], and also that price discounts significantly increase fruit and vegetable purchases [14]. More frequent promotions on fruit and vegetables in discount supermarkets could, therefore, greatly benefit lower SES groups. An increase in the frequency of promotions on healthy foods advertised in store flyers of discounters could encourage healthier eating in this particular group.

### Limitations

To our knowledge, this is one of the first studies to explore the healthiness of supermarket promotions. However, there are several limitations to our findings. Firstly, the supermarkets studied did not include those with the second- and third-largest market shares in the Netherlands. Two supermarkets with relatively large market shares of 7.9% and 5.5%, respectively, were excluded because they do not publish a weekly supermarket flyer, or only publish flyers that describe their product range. The exclusion of supermarkets could affect the generalizability of these results. However, our supermarket sample represented 59% of the total market, and included a range of different supermarket types and consumer segments. Moreover, the market shares of the supermarkets are very similar. A second limitation is the system of classification used to identify healthier and unhealthier product promotions. Within this system, we were unable to use the criterion for added sugar, since this is not provided on the nutritional values of product packages, nor is it included in the Dutch Food Composition Table. Nevertheless, these missing values could mostly be accounted for by referring to the energy content (e.g., a higher level of sugar increases the level of energy).

Another limitation was that the advertisements were assessed at the level of promotion rather than product. By using this system, promotions were categorized into one specific healthiness category, even if the individual products within that promotion (different kinds of cheese, for example) fell into different healthiness categories. It was therefore possible for one promotion to include products from several healthiness categories. Such promotions were categorized into the lowest category of healthiness, regardless of the size of the distribution of healthy and unhealthy products, making it possible for promotions that contained mainly healthy products to be assigned to the rare group. An example would be a promotion on bread, for which consumers are able to choose between white bread (rare) and whole wheat bread (preference). Since the lowest level of health in this promotion was rare, the overall promotion was assigned to that group. Sensitivity analysis revealed no significant differences in percentage of discount when bundled promotions were re-classified from the unhealthier to the healthier category, and most of the promotions can still be seen as ‘unhealthy’. The reclassifying of the promotions reduced the differences between the percentages of discounts (28.4% to 25.9%), although the differences in percentage of discount were still significant (p < .001). This classification strategy was chosen because a promotion including both healthy and unhealthy products did not make the healthy choice the easy choice for the consumer [47,48].

This study does not show the proportion of the total supermarket product range which is healthy or unhealthy, nor which proportion of each is promoted. For example, if healthy foods account for only a small proportion of the total range of products, the observed promotion level could be relatively high. If that is the case, it would be interesting to know how consumers would react to a product range with a higher proportion of healthier products that are also more frequently discounted.

## Conclusion

This comprehensive cross-sectional study has yielded important new findings on the proportion of healthy food promotions advertised through store flyers. The results of this study revealed that unhealthy promotions are advertised in store flyers more often than healthy promotions. Moreover, consumers had to buy more products when the promotion was unhealthy, providing an extra incentive to buy more unhealthy products. Future research should explore the proportion of healthy and unhealthy food promotions in relation to the supermarket’s total product range, to reveal whether there are not only more unhealthy products on special but more unhealthy products overall.
